# Crude* Aloe vera* Gel Shows Antioxidant Propensities and Inhibits Pancreatic Lipase and Glucose Movement* In Vitro*

**DOI:** 10.1155/2016/3720850

**Published:** 2016-01-03

**Authors:** Urmeela Taukoorah, M. Fawzi Mahomoodally

**Affiliations:** Department of Health Sciences, Faculty of Science, University of Mauritius, 230 Réduit, Mauritius

## Abstract

*Aloe vera* gel (AVG) is traditionally used in the management of diabetes, obesity, and infectious diseases. The present study aimed to investigate the inhibitory potential of AVG against *α*-amylase, *α*-glucosidase, and pancreatic lipase activity* in vitro*. Enzyme kinetic studies using Michaelis-Menten (*K*_*m*_) and Lineweaver-Burk equations were used to establish the type of inhibition. The antioxidant capacity of AVG was evaluated for its ferric reducing power, 2-diphenyl-2-picrylhydrazyl hydrate scavenging ability, nitric oxide scavenging power, and xanthine oxidase inhibitory activity. The glucose entrapment ability, antimicrobial activity, and total phenolic, flavonoid, tannin, and anthocyanin content were also determined. AVG showed a significantly higher percentage inhibition (85.56 ± 0.91) of pancreatic lipase compared to Orlistat. AVG was found to increase the Michaelis-Menten constant and decreased the maximal velocity (*V*_max_) of lipase, indicating mixed inhibition. AVG considerably inhibits glucose movement across dialysis tubes and was comparable to Arabic gum. AVG was ineffective against the tested microorganisms. Total phenolic and flavonoid contents were 66.06 ± 1.14 (GAE)/mg and 60.95 ± 0.97 (RE)/mg, respectively. AVG also showed interesting antioxidant properties. The biological activity observed in this study tends to validate some of the traditional claims of AVG as a functional food.

## 1. Introduction

*Aloe vera *is one of nature's most sacred therapeutic medicinal food plants. The medicinal potential of this tropical succulent has urged recurrent myths about its properties that have persisted from the fourth century BC throughout world history [1]. There are also reports that tend to show that Alexander the Great used* Aloe vera* to treat his wounded soldiers and Cleopatra used it for skin care [2]. Over the years, this exquisite plant has acquired names such as “the wand of heaven,” “heaven's blessing,” and “the silent healer” [3]. Native to Northern Africa, this plant has been in existence for over 2000 years [2].

Parenchymatous gel from* Aloe vera *leaves is extensively used in folk medicine, health drinks, topical creams, toiletries, and cosmetics [4]. Without any doubt, the commercialisation of* Aloe vera *is a success story. Various kinds of natural-based industries have a share in the* Aloe vera* market, most notably the cosmetic, food, beverage, and dietary supplement industries. The major constituents of* Aloe vera* gel can be classified into five groups, namely, phenolics, saccharides, vitamins, enzymes, and low molecular weight substances [5].* Aloe vera* gel has an assortment of pharmacological properties which encompasses antiviral, antibacterial, laxative, protection against radiation, antioxidant, anti-inflammation, anticancer, antidiabetic, antiallergic, and immunostimulation properties amongst others [5, 6].

Apart from skin disorders,* Aloe vera* gel can also be also applied on superficial or partial thickness burns to fasten healing process and reduce pain [7, 8]. It helps in soothing skin injuries affected by burning, skin irritations, cuts, and insect bites. Faster wound closure has also been demonstrated in rats treated with isolated and characterised* Aloe vera* polysaccharides [9].* Aloe vera* further reduces inflammation by downregulating proinflammatory cytokine production in activated human macrophages and thus interfering with the cytokine overproduction during early sepsis or in chronic inflammatory or autoimmune disease, thereby ameliorating the outcome and quality of life of patients [10].* Aloe vera* polysaccharides have also been speculated to enhance immunity activity and exert antioxidant effects in oral ulcer animal models [11].

Furthermore,* Aloe vera* has shown its potential in the management of diabetes mellitus (DM). Clinical trials have shown that, in obese individuals with prediabetes or early untreated diabetes mellitus,* Aloe vera *gel complex reduced body weight, body fat mass, and insulin resistance [12]. Abo-Youssef and Messiha (2013) also proved the antidiabetic effect of* Aloe vera* leaf pulp extract* in vivo* and* in vitro* as compared to glimepiride [13–16].

Despite the availability of panoply of information on* Aloe vera*, there is insufficient scientific evidence on the efficacy of untreated or unprocessed local cultivar of Mauritian* Aloe vera* gel and its mechanism of action. Additional work is required to probe into the antidiabetic, antimicrobial, and antioxidant properties of the Mauritian* Aloe vera* gel which may help to validate its traditional claims. This endeavour will also delineate further health benefits of* A. vera *so as to encourage its use as a herbal medicine or functional food. Therefore, the main aim of this* in vitro* study was to investigate the antidiabetic, antimicrobial, and antioxidant activities of crude* Aloe vera* gel.

## 2. Methodology

### 2.1. Plant Material

**Figure 1:**
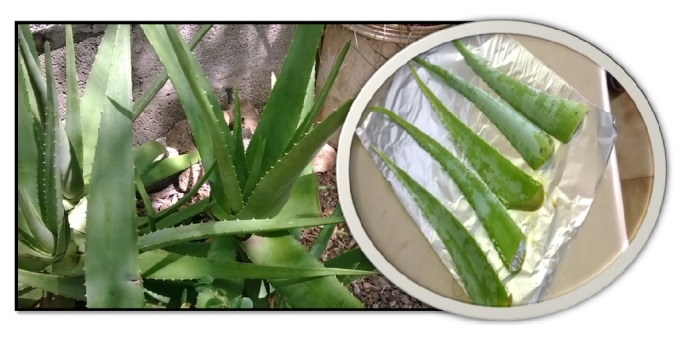
Freshly cut* Aloe vera* leaves.

Fresh* Aloe vera* leaves (Figure 1) were collected from Vacoas in October 2013. They were authenticated and an identification code (UTAV201301) was assigned to the specimen.

### 2.2. Preparation of Plant Material

The leaves were washed thoroughly under running tap water and then patted dry using clean filter papers. The peels were discarded while the gel was finely crushed using an electric blender. The resulting gel paste was used in all the tests.

### 2.3. Alpha-Amylase Inhibition Assay

The activity of *α*-amylase was carried out according to the method of Mao and Kinsella [17], based on the starch-iodine colour changes with minor modifications. Soluble starch solution (1%) was used as substrate. The starch solution was prepared by adding 1 g of soluble potato starch in 10 mL water and then boiled for 2 minutes. After cooling, water was added to reach a final volume of 100 mL. *α*-amylase solution (0.1 mL of 15 *μ*g/mL in 0.1 M acetate buffer at pH 7.2 containing 0.0032 M sodium chloride) was added to a mixture of 3 mL of 1% soluble starch solution and 2 mL of acetate buffer (0.1 M, pH 7.2) preequilibrated at 30°C in a water bath. Substrate and *α*-amylase blank determinations were undertaken under the same conditions.

At zero time (*t* = 0 min) and at the end of the incubation period (*t* = 60), 0.1 mL of reaction mixture was withdrawn from each tube after mixing and transferred into 10 mL of an iodine solution (0.254 g iodine and 4.0 g potassium iodide in 1 litre). After mixing, the absorbance of the starch-iodine mixture was measured immediately at room temperature at 565 nm using a spectrophotometer. The absorbance of the starch blank was subtracted from the sample reading. One unit of amylase activity was arbitrarily defined as follows: (*A*_*o*_ − *A*_*t*_/*A*_*o*_)*∗*100, where *A*_*o*_ and *A*_*t*_ are absorbance of the iodine complex of the starch digest at zero time and after 60 minutes of hydrolysis. Specific activity of *α*-amylase was defined as units/mg protein/60 min. Percentage inhibition was calculated using the following equation:

(1) %  inhibition=Ao−Atcontrol−Ao−AtsampleAo−Atcontrol∗100.

### 2.4. Alpha-Glucosidase Inhibition Assay

Alpha-glucosidase inhibitory activity was performed following the modified method of Pistia-Brueggeman and Hollingsworth [18]. In test-tubes, a reaction mixture containing 500 *μ*L of phosphate buffer (50 mM, pH 6.9), 100 *μ*L of *α*-glucosidase (1 U/mL), and 200 *μ*L of plant extract of varying concentrations was preincubated for 5 minutes at 37°C and then 200 *μ*L of 1 mM PNPG (4-nitrophenyl-alpha-D-glucopyranoside) substrate was added to the mixture. After further incubation at 37°C for 30 minutes, the reaction was stopped by the addition of 500 *μ*L of sodium carbonate (0.1 M). Enzyme, inhibitor, and substrate solutions were all prepared using the same buffer. Acarbose (5 mg/mL) was used as a positive control and water as a negative control. The yellow colour produced (due to* p*-nitrophenol formation) was quantitated by colorimetric analysis and reading the absorbance at 405 nm. Each experiment was performed in triplicate, along with appropriate blanks. The percentage inhibition was calculated using the formula

(2) $$\begin{array}{c} \%\;\;inhibition=\left[\;\frac{\left(A_{control}-A_{sample}\right)}{A_{control}}\;\right]*100. \end{array}$$

### 2.5. Porcine Pancreatic Lipase Inhibitory Assay

The porcine pancreatic lipase inhibitory assay was adapted from Zheng et al. and Bustanji et al. [19, 20]. Briefly, 50 *μ*L of porcine pancreatic lipase (1 mg/mL) was added to a mixture containing 100 *μ*L extract and 50 *μ*L tris-HCl buffer (2.5 mM, pH 7). The resulting mixture is then incubated at 37°C for 15 minutes. After the incubation period, 100 *μ*L of PNPB is then added to the test-tube. The mixture is again incubated for 1 h at 37°C. The absorbance is read at 405 nm. Percentage inhibition is calculated using the equation below:

(3) $$\begin{array}{c} \%\;\;inhibition=\left[\;\frac{\left(A_{control}-A_{blank}\right)}{A_{blank}}\;\right]*100. \end{array}$$

### 2.6. Enzyme Kinetics Using Michaelis-Menten and Lineweaver-Burk Equations

The assay was adapted from Zheng et al. [19] and* p*-nitrophenol calibration curve was generated. The extract and porcine pancreatic lipase were mixed in a ratio of 2 : 1, respectively, and preincubated for 15 minutes. After 15 minutes, 150 *μ*L of enzyme-extract mixture was added to each well containing 50 *μ*L buffer and 100 *μ*L PNPB of varying concentrations (with starting concentration 25.5 mM). The plate was then put in the incubator at 37°C for 30 minutes. After the incubation period, absorbance was determined at 405 nm. A double reciprocal plot using Michaelis-Menten and Lineweaver-Burk equations was generated.

### 2.7. Effect of* Aloe vera* Gel on* In Vitro* Glucose Movement

*In vitro* glucose diffusion studies were carried out by following the method with slight modifications described by Gallagher et al. and Edwards et al. [21, 22]. Briefly, the system consisted of a one-sided sealed dialysis tube (10 cm × 15 mm, dialysis tubing membrane, Sigma-Aldrich MW12173) into which 2 mL of 22 mM D-glucose in 0.15 M NaCl and 1 mL extract (50 g/L)/control (water) were incorporated. The other end was then sealed and the membrane was placed into a 100 mL glass beaker containing 40 mL 0.15 M NaCl and 10 mL distilled water to equilibrate the strength of internal and external medium. The beakers were placed into an orbital shaking incubator (JISICO) at 37°C at 100 rpm. The movement of the glucose into the external medium with respect to a negative control was monitored at set time intervals, that is, 0, 1, 2, 3, and 4 hrs. The absorbance was read at 500 nm. All tests were carried out in triplicate and glucose concentrations were measured using glucose oxidase kit method (GIBCO, Italy).

### 2.8. Antimicrobial Assay

The antibacterial and antifungal properties of the extracts were assessed by using modified antimicrobial assay utilising microtitre plate described by Drummond and Waigh [23]. Briefly, using aseptic techniques a single colony of microbe was transferred into a 100 mL bottle of peptone water broth, capped, and placed in incubator overnight at 35°C. After 12–18 h of incubation, using aseptic preparation and the aid of a centrifuge, a clean sample of microbe was prepared. To the sterile microtitre plates, 100 *μ*L of peptone water broth is first added to all wells, followed by 100 *μ*L of extract or control. Then, 100 *μ*L of microbial culture was further added. The resulting mixtures were then left to incubate for 24 h at ambient temperature. After incubation, 40 *μ*L INT (iodonitrotetrazolium) (0.2 mg/mL) was added to all the wells and left to incubate for a further 20 minutes. The microplates were then assessed visually to determine the minimum inhibitory concentration (MIC). Controls used for bacteria are Gentamicin, Chloramphenicol, and Streptomycin while controls used for fungi are Ampicillin and Amphotericin.

### 2.9. Ferric Reducing Antioxidant Power (FRAP) Assay

The ability of the extracts to reduce iron was determined according to the modified method of Benzie and Strain [24] where Trolox was used as standard. Plant extracts of varying concentrations were mixed with 2850 *μ*L FRAP solution (25 mL acetate buffer (300 mM, pH 3.6), 2.5 mL 2-4-6 tripyridyl-s-triazine (10 mM in 40 mM hydrochloric) (TPTZ; Sigma-Aldrich, Sydney, Australia), and 2.5 mL hydrated ferric chloride solution (20 mM) previously equilibrated for 30 minutes in the dark at 37°C). The reaction was allowed to take place for 30 minutes in the dark. The absorbance was determined at 593 nm. All determinations were done in triplicate and data obtained were expressed as mM Trolox Equivalent (TE)/mg crude extract.

### 2.10. Nitric Oxide Radical Scavenging Assay

Nitric oxide (NO) was generated from sodium nitroprusside (SNP) (Sigma-Aldrich, Sydney, Australia) and was measured by the Griess Ilosvay reagent [25], using 0.1% w/v naphthylethylene-diamine-dihydrochloride (Sigma-Aldrich, Sydney, Australia) instead of 5% 1-naphthylamine. Plant extract (0.5 mL) was added to a mixture containing SNP (2 mL) and phosphate buffer saline (PBS) (0.5 mL, pH 7.4). The reaction mixture was incubated for 2.5 hrs at 25°C. Following incubation, 0.5 mL of the reaction mixture was added to 1 mL sulphanilic acid (0.33% in 20% glacial acetic acid) (Sigma-Aldrich, Sydney, Australia) and allowed to stand for 5 minutes. Naphthylethylene-diamine-dihydrochloride (1 mL of 0.1% w/v) was then added to the mixture. The resulting solution was vortexed and allowed to stand for further 30 minutes. The absorbance of the chromophores formed during the diazotization of nitrite with sulphanilamide and subsequent coupling with naphthylethylene-diamine-dichloride was read at 546 nm. Percentage inhibition was calculated as follows:

(4) $$\begin{array}{c} \%\;\;inhibition=\left[\;\frac{\left(A_{blank}-A_{sample}\right)}{A_{blank}}\;\right]*100. \end{array}$$

### 2.11. DPPH Free Radical Scavenging Assay

The free radical scavenging activity of the different extracts was measured by using 1,1-diphenyl-2-picrylhydrazyl (DPPH) (Sigma-Aldrich, Sydney, Australia) according to the modified method of Umamaheswari and Chatterjee [26]. Plant extract (100 *μ*L) was added to 200 *μ*L freshly prepared DPPH solution (100 *μ*M in methanol). The reaction mixture was incubated at 37°C for 30 minutes. After incubation, absorbance was determined at 517 nm. The percentage inhibition of DPPH was calculated using (4).

### 2.12. Xanthine Oxidase Inhibitory Activity

The xanthine oxidase (Sigma-Aldrich, Germany) inhibitory activity was determined using modified method of Abdullahi et al. [27]. Samples were assayed for their* in vitro* xanthine oxidase inhibitory activity which was evaluated spectrophotometrically using xanthine as the substrate. The assay mixture consisted of 1 mL of the fraction, 2.9 mL phosphate buffer (pH 7.5), and 2 mL xanthine (0.15 mM) prepared in buffer. The mixture was vortexed and left to stand for 15 minutes. After 15 minutes, 0.1 mL of xanthine oxidase enzyme solution (0.1 unit/mL in phosphate buffer, pH 7.5), prepared immediately before use, was added. The mixture was then incubated at ambient temperature for 30 minutes. After the incubation period, reaction was stopped by the addition of 1 mL of 1 M hydrochloric acid. The absorbance was measured at 290 nm using a UV spectrometer. Allopurinol (100 *μ*L/mL), a known inhibitor of XO, was used as a positive control. One unit of XO is defined as the amount of enzyme required to produce 1 mmol of uric acid/min at 25°C. XO inhibitory activity was expressed as the percentage inhibition of XO by using the following equation:

(5) $$\begin{array}{c} \%\;\;inhibition=\left[\;1-\left(\;\frac{B}{A}\;\right)\;\right]*100, \end{array}$$

where *A* represents the activity of the enzyme without plant extract and *B* is the activity of XO in the presence of plant extract.

### 2.13. Determination of Total Phenol, Flavonoid, and Anthocyanin Tannin Content

The total phenolic content was evaluated using the modified Folin-Ciocalteu assay described by Nickavar and Esbati [28]. The plant extract (0.50 mL) was added to a test-tube containing a tenfold diluted Folin-Ciocalteu reagent solution (2.50 mL) and sodium carbonate (2.00 mL, 7.5%). The mixture was allowed to react for 30 minutes at room temperature. The total phenolic content was then spectrophotometrically determined at 760 nm. All determinations were carried out in triplicate and results obtained were expressed as *μ*g gallic acid equivalent (GAE)/mg crude extract.

The total flavonoid content was evaluated according to the aluminium chloride colorimetric method [29]. The plant extract (2 mL) was added to 2% aluminium chloride solution (2 mL). The mixture was allowed to react for 30 minutes at room temperature and the absorbance was read at 420 nm. All determinations were performed in triplicate and results obtained were expressed as *μ*g rutin equivalent (RE)/mg crude extract.

The total anthocyanin content was calculated using the pH differential method [30]. Briefly, 1 mL of plant extract was transferred into 10 mL volumetric flask and the volume was adjusted with buffer pH 1.0 and pH 4.5. Mixtures were allowed to equilibrate for 15 minutes. Absorbance of each dilution was spectrophotometrically determined at 510 and 700 nm. Absorbance of diluted samples was evaluated using the following equation:

(6) $$\begin{array}{c} A={\left(\;A_{510}-A_{700}\;\right)}_{pH1.0}-{\left(\;A_{510}-A_{700}\;\right)}_{pH4.5}. \end{array}$$

The monomeric anthocyanin pigment concentration in the original sample was calculated according to the following equation:

(7) Anthocyanin  content  mg/mL=A∗MW∗DF∗1000ɛ∗1,

where MW is the molecular weight of cyanidin-3-glucoside (484.5), DF the dilution factor, and *ɛ* the molar extinction coefficient (26,900).

Quantitative estimation of tannin, as catechin equivalent, was evaluated using the vanillin-HCl method with slight modifications. Extract (1 mL) was added in 5 mL of reagent mix containing 4% vanillin (in methanol) and 8% concentrated hydrochloric acid (in methanol). The resulting reaction mixture was vortexed and kept in the dark for 20 minutes. The absorbance was then determined at 500 nm using a spectrophotometer, using catechin (400 *μ*g/mL) as standard.

### 2.14. Statistical Analysis

All data were expressed as means ± SD for 3 experiments. Statistical analyses were performed using statistical software, namely, SPSS version 21.0 for Windows 7 and Excel software (Microsoft 2010).

## 3. Results

### 3.1. Inhibitory Activity of* Aloe vera* Gel on Key Enzymes

Data obtained from the *α*-amylase inhibition assay (Table 1) indicated no enzyme inhibitory activity for the* Aloe vera* extract when compared to the positive control (Acarbose, 400 *μ*g/mL) which had percentage of inhibition (96.64 ± 0.09%). Results from *α*-glucosidase inhibition assay showed no enzyme inhibitory activity for the* Aloe vera* extract when compared to the positive control (Acarbose, 5 mg/mL).* Aloe vera* gel exhibits a significantly higher (*p* < 0.05) percentage inhibition of 85.56 ± 0.91% than the positive control (Orlistat, 0.48 mM) against pancreatic lipase.

### 3.2. Enzyme Kinetic Studies

**Figure 2:**
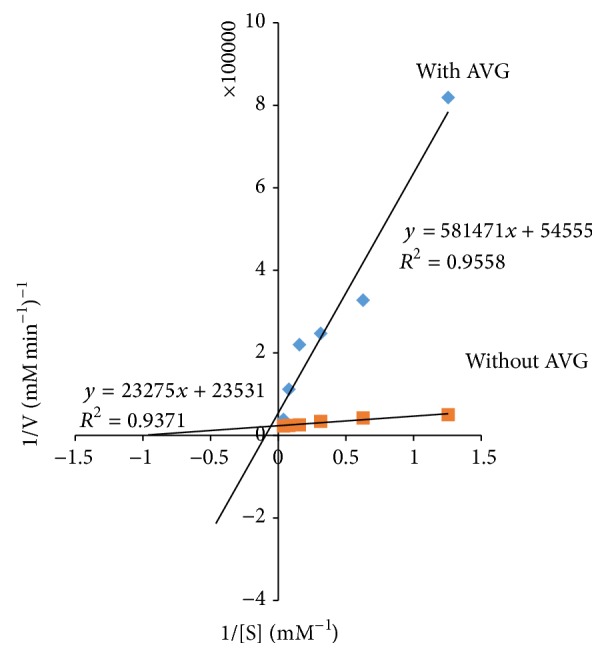
The Lineweaver-Burk plots in the presence and absence of* Aloe vera* gel.

*Aloe vera* was further assessed through kinetic studies to determine the type of inhibition on pancreatic lipase (Figure 2). The Lineweaver-Burk plot was generated using the calibration curve of nitrophenol, mentioned in Section 2. The double reciprocal Lineweaver-Burk plots showed a decrease in maximal velocity (*V*_max_) and an increase in Michaelis-Menten constant (*K*_*m*_), thereby suggesting mixed inhibition. *V*_max_ decreases from (4.25 × 10^−5^) mM min^−1^ to (1.83 × 10^−5^) mM min^−1^ while *K*_*m*_ increases from 0.99 to 10.66.

### 3.3. Effect of* Aloe vera* Gel on Glucose Movement* In Vitro*

**Figure 3:**
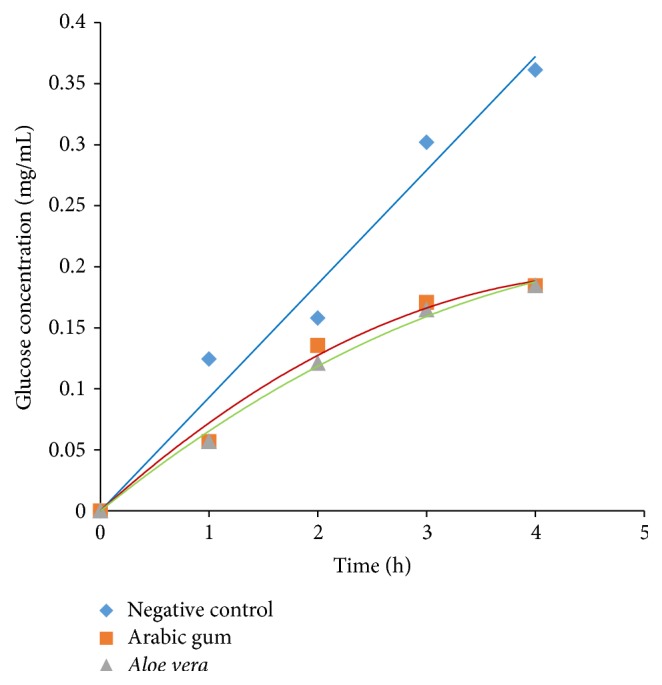
Glucose movement* in vitro.*

Figure 3 shows that* Aloe vera* mimics the action of Arabic gum (positive control) and thus retards glucose movement considerably. No significant difference (*p* > 0.05) was observed between the positive control and* Aloe vera* gel. The glucose content in dialysate and GDRI are depicted in Tables 2 and 3, respectively.

### 3.4. Antimicrobial Screening

*Aloe vera* gel did not inhibit the growth of any of the microbes tested (Table 4). However, when compared to the blank, the results showed a less intensive red colour for* Staphylococcus aureus*,* Escherichia coli*, and* Pseudomonas aeruginosa*. This tends to suggest that* Aloe vera* (though it did not inhibit bacterial growth) showed reduced growth. The controls used against bacteria are Streptomycin, Chloramphenicol, and Ciprofloxacin while the one used against fungi is Amphotericin.

### 3.5. Antioxidant Activities of* Aloe vera *Gel

In FRAP assay, the antioxidant activity was determined by generating the standard curve in the range of 0–400 *μ*M and the results were expressed as mM Trolox Equivalent (TE)/mg crude extract (*y* = 0.0022*x*, *R* ^2^ = 0.996).* Aloe vera* gel showed a value of 72.42 ± 0.95 mM Trolox Equivalent (TE).  Table 5 shows the percentage inhibition of* Aloe vera* in the different assays.* Aloe vera* gel exhibits significantly lower percentage inhibition against DPPH and NO^∙^ free radicals than the corresponding control (*p* < 0.05). It also inhibits xanthine oxidase at a significantly lower percentage than the control (*p* < 0.05).

### 3.6. Phytochemical Screening

Total phenolic content is reported as gallic acid equivalents by reference to standard curve (*y* = 0.0099*x* and *R* ^2^ = 0.9879) and was found to be 66.1 ± 1.14 mg GAE/g of extract. The total flavonoid content was 60.9 ± 0.97 *μ*g rutin equivalent (RE)/mL crude extract, respectively, by reference to standard curve (*y* = 0.0092*x* + 0.4896 and *R* ^2^ = 0.883).* Aloe vera* also showed a total tannin content of 21.1 ± 1.9 *μ*g catechin equivalent by reference to standard curve (*y* = 0.0003*x* and *R* ^2^ = 0.9903). No anthocyanin was found. The results are summarised in Table 6.

## 4. Discussion

In the present study, unprocessed* Aloe vera* gel was assessed in terms of its ability to inhibit key carbohydrate hydrolysing enzymes. No significant inhibition was found explaining that the antidiabetic property of* Aloe vera* gel may not be due to enzyme inhibition. This contradicts the findings of Abu Soud et al., where dried* Aloe vera* crude extract showed significant (more than 80%) *α*-amylase inhibitory activity [31–33]. These disparities can be explained based on the fact that different extraction method was used. Another study reported moderate *α*-amylase inhibitory activity with low percentage inhibition by* Aloe vera *extracts [33]. However, the previous study has used soxhlet apparatus where dried plant parts were finely crushed, powdered, and extracted with methanol [34]. Methanol and ethanol are more efficient than water in cell walls and seeds degradation which have nonpolar character and cause active ingredients to be released from cells [35]. The decrease in activity of aqueous extract can also be ascribed to the enzyme polyphenol oxidase, which degrades polyphenols in water extracts but is inactive in methanol and ethanol [35]. Additionally, ethanol was found to penetrate the cellular membrane more easily to extract the intracellular ingredients from the plant material [35].

Dialysis tubing technique is a simple model to evaluate the potential of soluble dietary fibres to retard the diffusion and movement of glucose in the intestinal tract [36, 37]. There are experimental evidences suggesting that the retardation of the nutrient flow into the external medium is an indication of the modulating effect of fibre on glucose absorption in the intestine [36]. Hence, this assay may help to evaluate the hypoglycaemic effect of a particular extract. The effects of plant extracts on glucose movement* in vitro* have gained much attention due to the fact that in recent years national and international diabetes associations have emphasised the need to increase fibre intake which interacts with food nutrients reducing absorption, especially postprandial glucose after carbohydrate-rich meals [38].

In the present study,* Aloe vera *gel proved to be an efficient agent to retard glucose movement across the dialysis tubing. It followed almost the same trend as Arabic gum, used as a control. As reported by Ahmed et al., the retardation in glucose diffusion might be attributed to the physical obstruction towards glucose molecules or the entrapment of glucose molecules within the fibre network [39]. Hence, it can be deduced that the viscous and mucilaginous nature of* Aloe vera* gel entrapped the glucose molecules preventing them from moving into the external solution. The results thus support the use of* Aloe vera* gel to maintain blood glucose level in diabetic patients. The diabetic activity could also be attributed to reduced intestinal absorption of postprandial glucose, in order to prevent high peaks of glucose in the blood. Most of the studies carried out on the antidiabetic properties of* Aloe vera* used homogenised or processed* Aloe vera* extract. To our best knowledge, no information concerning effect of* Aloe vera* gel (in its natural form) on antidiabetic activities has been published. Abo-Youssef and Messiha [13] reported the antidiabetic role of* Aloe vera* and explained that this could be due to the potent antioxidant potential of* Aloe vera*. Another study used aqueous extract of* Aloe vera* (100 g* Aloe vera* boiled with 200 mL distilled water and then cooled) which was fed to diabetic rats which in turn resulted in lowered blood glucose level [40].

A varying range of lipases, responsible for the catalysis of hydrolysis of ester bonds in triacylglycerols, are produced by the human body [41]. Some pharmacological obesity treatments, for example, Orlistat, function through specific, irreversible inhibition of gastrointestinal lipases, of which pancreatic lipase is the most biologically active and important one in healthy humans [41]. Serious adverse effects are commonly reported for Orlistat, including steatorrhoea, bloating, oily spotting, faecal urgency, and faecal incontinence that can affect up to 40% of patients [41]. Hence, a product which inhibits pancreatic lipase and lowers/eliminates the adverse effects of present treatment would be of substantial benefit to patients.

The present study attempted to probe into the hypolipidemic effect of* Aloe vera* gel through the inhibition of pancreatic lipase. The results showed that* Aloe vera* gel exhibited a significantly higher percentage of inhibition than the positive control Orlistat. Furthermore, kinetics parameters were evaluated from the double reciprocal plot for* Aloe vera* gel. The results showed an increase in the Michaelis constant (*K*_*m*_) and a decrease in maximal velocity (*V*_max_) of the reaction, suggesting a mixed inhibition of pancreatic lipase. This is a major type of inhibition that occurs when the inhibitor is capable of binding to both the free-enzyme and the enzyme-substrate complex. Generally, when the inhibitor binds favourably to the free-enzyme, mixed inhibition leads to an increase in *K*_*m*_ value while if it binds preferentially to the enzyme-substrate complex, *K*_*m*_ value is decreased. Both cases are accompanied by a decrease in *V*_max_. In the present study, an increase in *K*_*m*_ and a decrease in *V*_max_ were observed suggesting that* Aloe vera* gel has a greater affinity for the free-enzyme that is pancreatic lipase. This offers the advantage of not being affected by higher concentrations of substrate compared to Orlistat which acts as a competitive inhibitor [42]. Our result tends to support a study conducted by Kim et al. [43] which showed that the administration of processed* Aloe vera* gel lowered triacylglyceride levels in liver. Additionally, the plasma and histological examinations of periepididymal fat pad showed that processed* Aloe vera* gel reduced the average size of adipocytes. Choi et al. [12] showed reduced body weight, body fat mass, and insulin resistance in obese individuals treated with* Aloe vera*. Choudhary et al. also proved the efficacy of* Aloe vera* by showing significant reduction in blood glucose, lipid profile, and blood pressure of the diabetic patients [44]. According to the present study's results, it can be deduced that* Aloe vera *gel inhibits porcine pancreatic lipase and thus deserves to be further explored as a weight lowering agent to combat obesity.

The present study also aimed to investigate the antimicrobial attributes of* Aloe vera* gel. The extract was tested against 5 common microorganisms. Results clearly demonstrate that* Aloe vera* gel, in its natural form, does not inhibit microbial growth. This tends to contradict results obtained by Khaing, where he demonstrated that* Aloe vera* inhibited numerous human pathogens except* Candida albicans *[45]. The present results also contradicted the results obtained by Nejatzadeh-Barandozi [46], where* Aloe vera* suppressed the growth of* Staphylococcus aureus*,* Escherichia coli*, and* Pseudomonas aeruginosa* amongst others. The disparity can be explained by the use of ethanol extract which contains more bioactive components than the unprocessed* Aloe vera* gel. Hence, it can be deduced from preliminary data gathered in the present study that unprocessed* Aloe vera* gel is not an effective antimicrobial agent.

Reactive oxygen species (ROS) have potent oxidative effects on cellular constituents which can in turn impair cellular functions [47]. Therefore, they are associated with pathogenesis of insulin resistance via the inhibition of insulin signals and dysregulation of adipocytokines/adipokines which play an important role in the progression of diabetes, hypertension, atherosclerosis, and even cancer [48]. Scavenging of free radicals is believed to be a valuable measure to prevent or treat diseases like diabetes mellitus [49].

For the determination of antioxidant properties of* Aloe vera* gel, different antioxidant assays like DPPH, NO, FRAP, and xanthine oxidase inhibitory assay were performed.* Aloe vera* gel showed low DPPH and NO^∙^ scavenging abilities in the current study. Though significantly lower than the positive control, it is evident that the extract did show some proton-donating ability and could serve as free radical inhibitor or scavenger, acting possibly as antioxidants. The results however contradict the findings of Khaing [45]. The latter proved that* Aloe vera* had strong DPPH scavenging ability. The difference can be explained by the fact that 95% ethanol* Aloe vera* extract was used which has a higher percentage yield of bioactive ingredients. Yet, here also, the antioxidant activity was not as high as the positive controls. Another study compared the antioxidant activity of* Aloe vera* extracted in different solvents and obtained highest DPPH inhibition in methanol extract of* Aloe vera *[34]. The results also displayed xanthine oxidase inhibition. Xanthine oxidase is a form of xanthine oxidoreductase, a type of enzyme that generates ROS. These enzymes catalyze the oxidation of hypoxanthine to xanthine and can further convert the oxidation of xanthine to uric acid.* Aloe vera gel* also showed significant ferric reducing antioxidant power which adds up to its antioxidant properties. However, a study showed that* Aloe vera* extracts obtained from supercritical carbon dioxide extraction and ethanol showed stronger antioxidant activities than BHT and *α*-tocopherol [50].

Over the past 10 years, there has been a growing interest in the potential of polyphenols among researchers and food manufacturers mainly because of their antioxidant properties, their abundance in our diet, and their role in the prevention of various diseases associated with oxidative stress such as cancer, cardiovascular disease, and neurodegeneration [46]. Polyphenols are reputed for their antioxidant activities and these compounds are multifunctional and can act as reducing agents, hydrogen-donating antioxidants, and singlet oxygen quenchers. The current study showed that* Aloe vera* gel possesses significant amount of phenols and flavonoids which contribute to the antioxidant activities but contain little tannins and no anthocyanins. This support the findings of Patel et al. [51] which reported that the* Aloe vera* extract contained significant amount of phenol, flavonoids, berberine, and gallic acid through HPLC techniques. However, the crude* Aloe vera* extract used in the experiments was from a commercial source.

Therefore, the present* in vitro *study has provided sound scientific footing to enhance assurance on the traditional remedy of* Aloe vera, *which may be efficient as a preventive agent in the pathogenesis of some diseases. It is anticipated that data amassed in the present study will open new avenues for the development of potential drugs that can be used to treat and/or manage DM, obesity, and related complications.

## Figures and Tables

**Table 1:** Percentage inhibition of AVG against key enzymes.

Sample	Percentage inhibition		
	*α*-amylase	*α*-glucosidase	Pancreatic lipase
Acarbose	96.64 ± 0.10	62.70 ± 0.15	70.58 ± 0.50
*Aloe vera*	−4.88 ± 0.09	−0.81 ± 0.33	85.56 ± 0.91^*∗*^

^*∗*^ *p* < 0.05 compared to the control (Acarbose), one-way ANOVA.

**Table 2:** Glucose content in dialysate.

Sample	Glucose content in dialysate (mg/mL)				One-way ANOVA (*t* = 4)	
	1 hour	2 hours	3 hours	4 hours	*F*	*p* value
Arabic gum *Aloe vera*	0.057 ± 0.005 0.057 ± 0.005	0.136 ± 0.003 0.124 ± 0.003	0.171 ± 0.003 0.165 ± 0.005	0.185 ± 0.001 0.185 ± 0.005	1088.047	>0.05

**Table 3:** Glucose diffusion retardation index.

Sample	Glucose diffusion retardation index			
	1 hour	2 hours	3 hours	4 hours
Arabic gum	9954	9914	9943	9949
*Aloe vera *	9954	9923	9945	9949

**Table 4:** Antimicrobial activity of *Aloe vera*.

Antimicrobial strains	MIC (mg/mL)				
	*Aloe vera*	Streptomycin	Chloramphenicol	Ciprofloxacin	Amphotericin
*Staphylococcus aureus*	—	0.002	0.004	No growth	—
*Escherichia coli*	—	0.016	0.002	No growth	—
*Pseudomonas aeruginosa*	—	0.008	0.063	No growth	—
*Candida albicans*	—	—	—	—	0.063
*Candida tropicalis*	—	—	—	—	0.063

**Table 5:** Antioxidant activity of *Aloe vera*.

Assays	Percentage inhibition		One-way ANOVA	
	*Aloe vera*	Control	*F*	*p* value
DPPH free radical scavenging assay	17.11 ± 1.30	81.78 ± 0.24^a^	7153.206	<0.05
NO^∙^ scavenging assay	34.88 ± 0.52	87.25 ± 0.74^b^	9935.925	<0.05
Xanthine oxidase inhibitory assay	73.85 ± 2.02	85.94 ± 0.11^c^	106.801	<0.05

^a^Ascorbic acid (400 *μ*g/mL), ^b^ascorbic acid (2 mg/mL), and ^c^allopurinol (100 *μ*g/mL).

**Table 6:** Phytochemical content of *A*. *vera*.

Assays	*Aloe vera*
Total phenolic content	66.06 ± 1.14^a^
Total flavonoid content	60.95 ± 0.97^b^
Total tannin content	21.11 ± 1.92^c^
Total anthocyanin content	ND

^a^ *μ*g gallic acid equivalent (GAE)/mg, ^b^ *μ*g rutin equivalent (RE)/mg, and ^c^ *μ*g catechin equivalent; ND: not detected.
